# Sequencing Using a Two-Step Strategy Reveals High Genetic Diversity in the S Gene of SARS-CoV-2 after a High-Transmission Period in Tunis, Tunisia

**DOI:** 10.1128/Spectrum.00639-21

**Published:** 2021-11-10

**Authors:** Wasfi Fares, Kais Ghedira, Mariem Gdoura, Anissa Chouikha, Sondes Haddad-Boubaker, Marwa Khedhiri, Kaouthar Ayouni, Asma Lamari, Henda Touzi, Walid Hammemi, Zina Medeb, Amel Sadraoui, Nahed Hogga, Nissaf ben Alaya, Henda Triki

**Affiliations:** a Laboratory of Clinical Virology, Reasearch Laboratory Viruses Vectors and Hosts (LR20-IPT10), Institut Pasteur, University of Tunis-El Manar, Tunis, Tunisia; b Laboratory of Bioinformatics, Biomathematics and Biostatistics (BIMS), Institut Pasteur de Tunis (IPT), University of Tunis-El Manar, Tunis, Tunisia; c National Observatory for New and Emerging Diseases, Ministry of Health, Tunis, Tunisia; d Faculty of Pharmacy, University of Monastir, Monastir, Tunisia; e Faculty of Medicine, University of Tunis-El Manar, Tunis, Tunisia; Johns Hopkins Hospital

**Keywords:** COVID-19, SARS-CoV-2, whole-genome sequencing, VOCs, VOIs, spike protein, Tunisia

## Abstract

Recent efforts have reported numerous variants that influence severe acute respiratory syndrome coronavirus 2 (SARS-CoV-2) viral characteristics, including pathogenicity, transmission rate, and detectability by molecular tests. Whole-genome sequencing based on next-generation sequencing technologies is the method of choice to identify all viral variants; however, the resources needed to use these techniques for a representative number of specimens remain limited in many low- and middle-income countries. To decrease sequencing costs, we developed a primer set allowing partial sequences to be generated in the viral S gene, enabling rapid detection of numerous variants of concern (VOCs) and variants of interest (VOIs); whole-genome sequencing is then performed on a selection of viruses based on partial sequencing results. Two hundred one nasopharyngeal specimens collected during the decreasing phase of a high-transmission COVID-19 wave in Tunisia were analyzed. The results reveal high genetic variability within the sequenced fragment and allow the detection of first introductions in the country of already-known VOCs and VOIs, as well as other variants that have interesting genomic mutations and need to be kept under surveillance.

**IMPORTANCE** The method of choice for SARS-CoV-2 variant detection is whole-genome sequencing using next-generation sequencing (NGS) technologies. Resources for this technology remain limited in many low- and middle-income countries, where it is not possible to perform whole-genome sequencing for representative numbers of SARS-CoV-2-positive cases. In the present work, we developed a novel strategy based on a first partial Sanger screening in the S gene, which includes key mutations of the already known VOCs and VOIs, for rapid identification of these VOCs and VOIs and to help better select specimens that need to be sequenced by NGS technologies. The second step consists of whole-genome sequencing to allow a holistic view of all variants within the selected viral strains and confirm the initial classification of the strains based on partial S gene sequencing.

## INTRODUCTION

Severe acute respiratory syndrome coronavirus 2 (SARS-CoV-2), which is the causative agent of human coronavirus disease 2019 (COVID-19), was identified in Wuhan, China, in December 2019 (1, 2). The COVID-19 outbreak rapidly spread worldwide; it was officially declared a pandemic by the World Health Organization (WHO) on 11 March 2020 (3) and now represents a tremendous threat globally.

SARS-CoV-2 is a single-stranded positive RNA virus, a member of the *Betacoronavirus* genus that also contains SARS-CoV and Middle East respiratory syndrome coronavirus (MERS-CoV). The first sequence of the virus was published in January 2020 (4). The structural genome region, located in the 3′ part of the genome, encodes four structural proteins: spike (S), envelope (E), membrane (M), and nucleocapsid (N) (5). The S protein forms a trimer on the surface of the virion; it mediates virus attachment to the angiotensin-converting enzyme 2 (ACE2) receptor and its entry to the host cells (6). The S protein is composed of two subunits, S1, containing the receptor-binding domain (RBD), and S2, which mediates membrane fusion (7). The S protein determines SARS-CoV-2 infectivity and transmissibility and is also the major antigen inducing a protective immune response (8). Since the beginning of the COVID-19 pandemic, the S protein has undergone several mutations, and it is highly important to follow the emergence of these variants and their biological, epidemiological, and clinical significance. Early in the pandemic, variants of SARS-CoV-2 containing a D-to-G substitution in residue 614 of the S protein (D614G) were reported. This substitution increased the receptor binding avidity, and D614G mutants became dominant in many geographic regions (9–11). In December 2020, the United Kingdom reported a variant of concern (VOC), referred to as the Alpha variant (B.1.1.7), with enhanced transmissibility within the population (12, 13). This variant became predominant in the United Kingdom and spread to more than 100 countries around the world. In January 2021, two other VOCs, referred as the Beta (B.1.351) and Gamma (B1.1.28) variants, also with high transmissibility, were reported in South Africa and Brazil, respectively (14–16). Later, many other variants, classified as variants under investigation (VUIs), were reported throughout the world. In addition to the increased transmissibility, it is suggested that some mutations in these variants may affect the performance of some diagnostic real-time PCR tests and reduce susceptibility to vaccine-induced neutralizing antibodies (9, 10, 17–22). Global tracking of these newly identified VOCs and VUIs, as well as any other evolving SARS-CoV-2 variants, by genomic surveillance and rapid sharing of viral genomic sequences is highly recommended in order to limit their spread and control the pandemic.

At present, several classifications of SARS-CoV-2 strains in lineages or clades have been proposed. Indeed, two different lineages, A and B, were proposed by the Phylogenetic Assignment of Named Global Outbreak (PANGO) lineage nomenclature, while a classification into 11 different clades (19-A, 19-B, and 20-A to 20-I) was proposed by the Nextstrain resources and another classification into 9 clades (S, L, O, V, G, GH, GR, GRY, and GV) was proposed by Global Initiative on Sharing All Influenza Data (GISAID).

In Tunisia, the first case of SARS-CoV-2 infection was reported on 3 March 2020 (23). The country experienced a first wave of the coronavirus disease and, through setting up drastic nationwide multisectoral measures to avoid international introduction of the virus and its spread within the population, COVID-19 incidence decreased in May to June 2020 to reach zero cases per day from the 4th to the 11th of June 2020. The national strategy included early detection of imported cases, quarantining of new confirmed cases and suspected cases, and strict travel restrictions. After the sharp decrease of the disease incidence, a relaxation in the application of these measures by the general population, combined with decreased restrictions in international transportation, led to the reintroduction of the virus again and the establishment of local transmission. In late July, the COVID-19 incidence started to increase again and the country experienced a second wave with the highest incidence in January 2021, associated with high local transmission within the population. Starting from February 2021, the disease incidence decreased, together with mortality rates, but the country experienced further waves of COVID with higher transmission rates. The last wave started in June 2021 with the introduction of the Delta variant and is in its decreasing phase at the time of writing.

The present work reports the genomic features of SARS-CoV-2 sequences detected in Tunisia during the late phase of the second wave of the pandemic using partial sequencing in the S gene followed by whole-genome sequencing of selected samples. This approach allowed the detection of several variants, some of which are already known as VOCs.

## RESULTS

**FIG 1:**
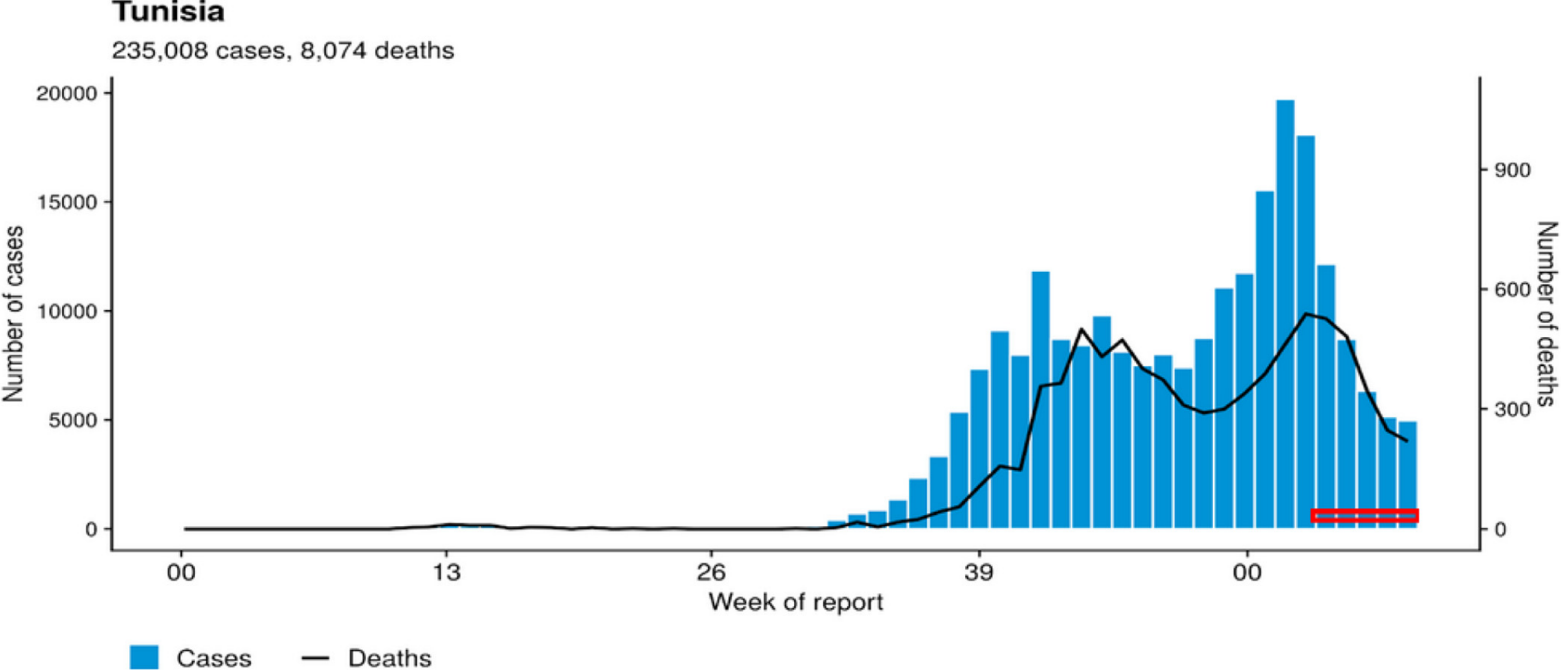
Sample collection period investigated in the present study. The graph displays the numbers of cases and the numbers of deaths in Tunisia since the declaration of the pandemic in March 2020. The *x* axis represents the number of weeks from March 2020 until May 2021. Weeks highlighted in red represent the sample collection period investigated in the present study.

A phylogenetic tree was constructed based on the alignment of a 659-nucleotide fragment in the S gene of the 201 studied Tunisian SARS-CoV-2 strains, together with the 9 selected reference SARS-CoV-2 sequences, according to the GISAID nomenclature (Fig. 1). The tree topology shows that the Tunisian sequences are divided into 3 different clusters. Cluster 1, represented by purple in Fig. 1, includes the highest number of sequences (174 out of 201, 86.5% of Tunisian strains) that clustered with the 4 reference sequences of the GISAID clades G, GH, GR, and GV. The phylogenetic distribution within this cluster shows several phylogenetic subbranches, reflecting a large genetic variability. Cluster 2, indicated with blue in Fig. 1, comprises 15 identical sequences that clustered with the GISAID reference sequence from clade GRY. Cluster 3, indicated with red in Fig. 1, contains 12 sequences that clustered with the GISAID reference sequence from clade S.

**FIG 2:**
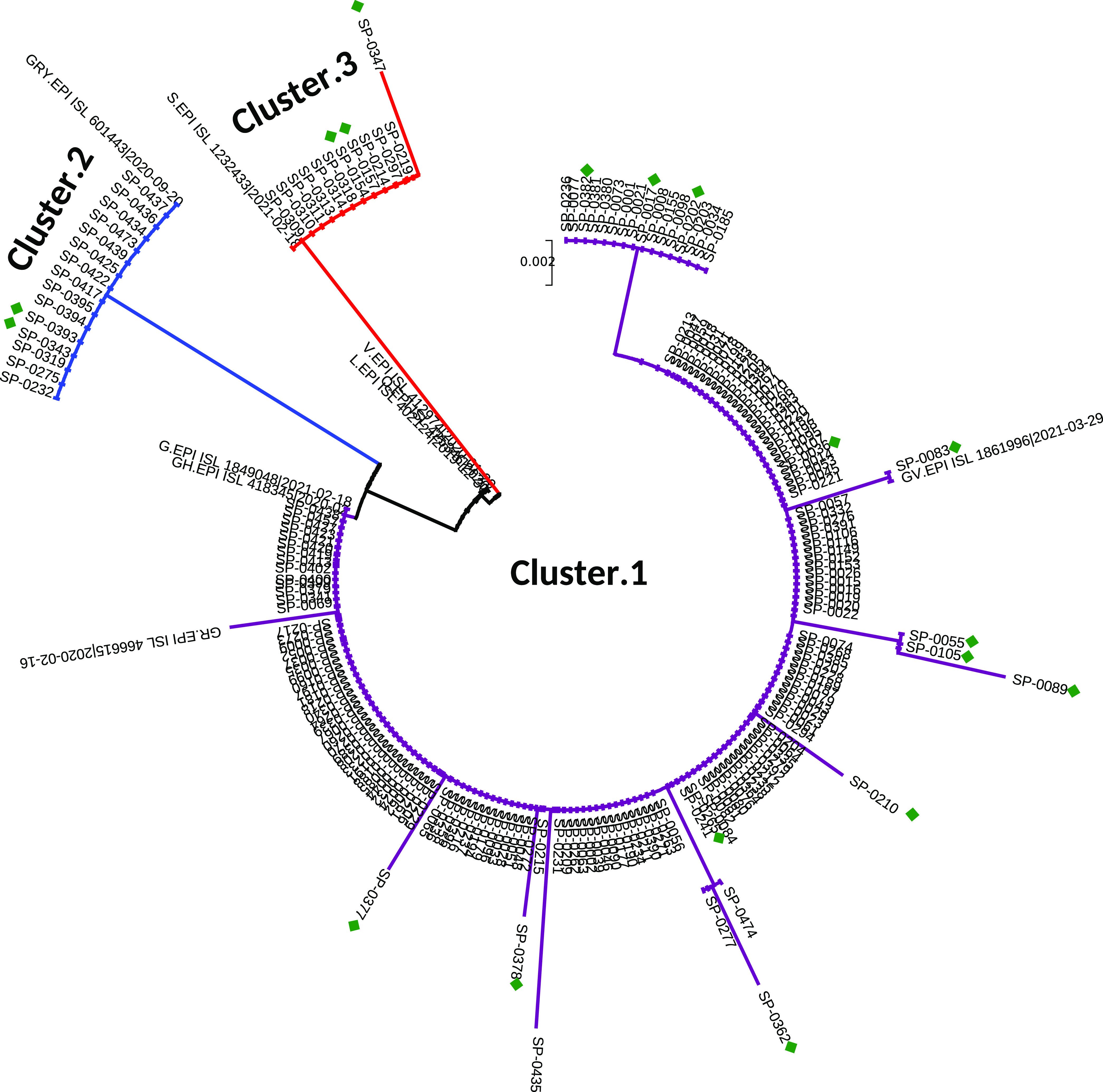
Phylogenetic tree of 201 SARS-CoV-2 sequences based on partial S gene nucleotide sequencing. The phylogenetic tree includes 201 Tunisian sequences compared to 9 representative reference sequences of SARS-CoV-2 clades. The tree was constructed using the neighbor-joining method and the Tamura 3-parameter (T92) model. Topology was supported by 1,000 bootstrap replicates. The sequences reported in this study are indicated by the laboratory code. The sequences downloaded from GISAID are indicated by their accession numbers. Cluster 1, marked in purple, includes sequences presenting the D614G substitution and the lack of the amino acid substitution N501Y. Cluster 2, marked in blue, includes sequences having the N501Y, A570D, D614G, and P681H substitutions. Cluster 3, marked in red, groups sequences with the N501Y, A653V, and H655Y substitutions and lacking the amino acid substitution D614G.

Eighteen representative samples from these clusters, indicated by green diamonds in Fig. 1, were selected for whole-genome sequencing: 13 from cluster 1, 2 from cluster 2, and 3 from cluster 3. The phylogenetic tree of the 18 whole-genome sequences obtained, together with the 9 GISAID SARS-CoV-2 reference sequences, is shown in Fig. 2. The figure also shows the classification of the Tunisian sequences according to the PANGO and the Nextstrain classifications. The phylogenetic distribution of the sequences based on whole-genome sequences (Fig. 2) is similar to the one shown in Fig. 1 that is based on the partial S gene genomic data.

The 13 sequences from cluster 1 highlighted with purple in Fig. 1 grouped together within the PANGO B lineage in Fig. 2. The phylogenetic distribution of these sequences clearly shows the presence of 3 subclusters, called subclusters 1a, 1b, and 1c and classified as clade G/20A, GV/20A-C and GH/20C, respectively, according to the GISAID/Nextstrain nomenclatures. Subcluster 1a is represented by only one sequence (SP-0362), while subcluster 1b and subcluster 1c are represented by 4 (SP-0202, SP-0083, SP-0377, and SP-0036) and 8 (SP-0378, SP-0017, SP-0382, SP-0210, SP-0084, SP-0089, SP-0055, and SP-0105) sequences, respectively.

Two sequences from cluster 2 presented in Fig. 1 were also found to cluster together with the reference sequence of the GISAID GR clade based on whole-genome sequencing comparison; the sequences also belong to the PANGO B lineage and to the 20B clade of the Nextstrain nomenclature.

Unlike the sequences from cluster 1 and cluster 2, the three whole-genome sequences from cluster 3 belonged to the PANGO A lineage. They grouped together with the reference sequence of the GISAID S clade, similar to the results obtained based on the partial S sequences.

The amino acid sequences related to the 201 partial S sequences and the 18 whole-genome sequences were deduced from the obtained nucleotide sequences and compared to the Wuhan reference protein sequences.

Table 1 shows the amino acid substitution profile in the sequenced fragment of the S gene of the 201 samples investigated in the present study. Fourteen different mutation profiles were found. Most of the sequences (147/174) had zero nonsynonymous mutations compared to the Wuhan reference virus, except for D614G, which was found in all the sequences from cluster 1 and cluster 2. The remaining 27 sequences from cluster 1 had either 1 or 2 additional substitutions within the sequenced fragment (Table 1). The 15 sequences from cluster 2 shared an identical mutational profile, containing the amino acid substitutions N501Y, A570D, D614G, and P681H that are known characteristics of the VOC Alpha (B.1.1.7) initially detected in the United Kingdom (12, 13). The 12 sequences from cluster 3 did not have the D614G substitution but had three mutations that suggest the VUI A.27 (N501Y, A653V, and Q655H); one sequence (SP-0347, which was in a separate branch within the phylogenetic tree shown in Fig. 1), had an additional substitution (Q677H).

**TABLE 1:** Amino acid substitution profile in the sequenced fragment of the S gene of the samples investigated in the present study

Mutation profile	No. of samples with mutation(s) in cluster:		
	1 (*n* = 174)	2 (*n* = 15)	3 (*n* = 12)
E484K, D614G	2	0	0
E484K, D614G, Q677H	1	0	0
D614G, S637L, A647S	1	0	0
D614G, I666L	1	0	0
D614G, Q675L	1	0	0
D574Y, D614G, A626S	1	0	0
D614G, A626S	2	0	0
D614G, V622F	1	0	0
D614G, E619Q	1	0	0
D614G, D627E	16	0	0
D614G	147	0	0
N501Y, A570D, D614G, P681H	0	15	0
N501Y, A653V, Q655H	0	0	11
N501Y, A653V, Q655H, Q677H	0	0	1

Table 2 shows the amino acid substitution profile along the whole genome of the 18 selected Tunisian SARS-CoV-2 samples and representatives from the different clusters found based on S partial sequences. Amino acid substitutions located in the targeted partial region of the S gene and previously found by partial sequencing are marked with a star in Table 2. The two sequences from cluster 2 had identical mutational profiles in the S gene and totals of 23 and 24 amino acid substitutions along the whole genome; these results confirm the assignment of the two sequences to the Alpha lineage (VOC). The three sequences from cluster 3 shared 15 identical amino acid substitutions along the whole genome, and the results confirm the assignment of the three sequences to the A.27 lineage, identified as a variant of interest (VOI) and initially detected in France. Among cluster 1, one sequence (SP-0062, in subcluster 1a) had a mutational profile that corresponded to the identified variant of interest Eta (B.1.525), initially detected in Nigeria and in the United Kingdom. The sequences from subcluster 1c shared several identical mutations in the nonstructural regions of the genome and belonged to the B.1.160 lineage that is not presently identified as a VOC or VOI. The same is true for the sequences from subcluster 1b that had more genetic diversity and belonged to the B.1.177 lineage.

**TABLE 2:**
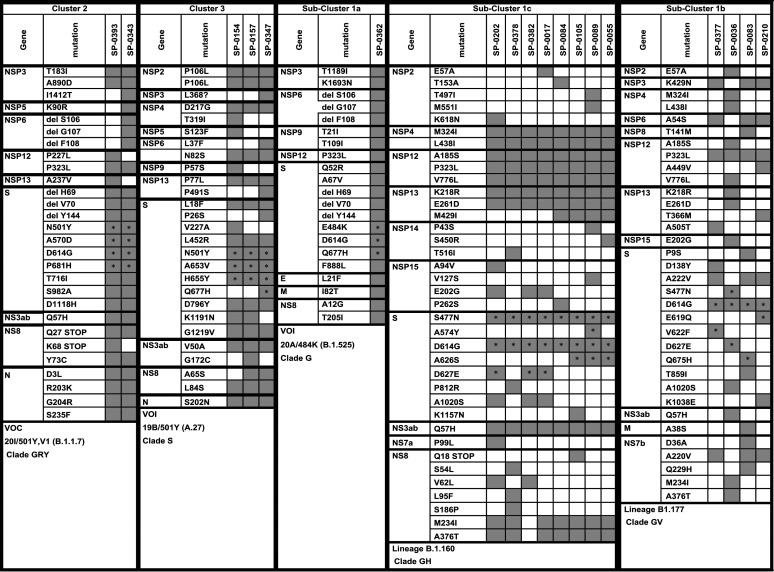
Mutation profile by WGS^*a*^

a Amino acid substitutions previously found by partial sequencing in the S gene are marked with a star.

## DISCUSSION

Since the beginning of the COVID-19 pandemic, several SARS-CoV-2 variants have emerged, and some of them totally changed the infection epidemiology. First, a variant with the D614G mutation emerged and became dominant globally (24). In our series, this mutation is found in 186 of the 201 isolates (92%). Other variants emerged subsequently, and it is now hypercritical to track those already labeled as VOCs or VOIs and also to monitor the emergence of new variants. The method of choice is whole-genome sequencing using next-generation sequencing (NGS) high-throughput technologies, which have improved considerably during recent years along with costs that have declined continuously. The cost of a whole-genome sequence depends on the type of machine and the workload in the sequencing laboratory and can be as expensive as and up to 10 times more expensive than Sanger sequencing, depending on the circumstances. Thus, access to NGS and resources for this technology remain limited in many low- and middle-income countries, where it is not possible to perform whole-genome sequencing for representative numbers of SARS-CoV-2-positive cases and where the use of other technologies to identify isolates that are to be sequenced as a priority is greatly needed. Several real-time PCR tests that target the already-known VOCs, especially the Alpha (United Kingdom), Beta (South Africa), and Gamma (Brazil) VOCs, are now commercially available. They can be very useful to rapidly identify the introduction of these VOCs to a country or region and to monitor their transmission. However, these kits cannot detect other variants of interest that have already emerged or that may emerge at any time. Furthermore, other variants can be characterized by the failure to detect the S gene in these tests, known as S gene target failure (SGTF) (25).

In the present work, we developed a primer set allowing a 659-nucleotide-long sequence in the viral S gene to be generated that includes key mutations of the VOCs and VOIs already known at the time of writing. Sequencing of this fragment by the traditional Sanger technology allows rapid identification of these VOCs and VOIs and helps to better select specimens that need to be sequenced by NGS technologies. Using this approach, it is possible to detect at least 16 amino acid substitutions that have been identified in almost all VOCs and VOIs (T478K, G482V, E484K, N501Y, A570D, D574Y, D614G, E619Q, A626S, D627E, A653V, H655Y, Q675H, Q677H, P681H, and P681R) and to get a rapid orientation toward an already known or a new variant. All of the amino acid substitutions that we detected by partial sequencing in the S gene were confirmed by WGS (marked with a star in Table 2), with total agreement between the two techniques.

In our series and using these primers, we were able to detect the first introduction of the B.1.1.7 variant (a VOC) and two other VOIs (A.27 and Eta) and to select other viruses for WGS, based on the results obtained in the partial S genomic region. The second step, consisting of whole-genome sequencing, confirmed the initial classification of the strains based on partial S gene sequencing and gave a holistic view of all mutations within the selected viral strains. In our study, 18 samples representative of the different clusters found by partial sequencing were assessed by WGS to compare the two approaches. However, depending on the access to WGS and for more representativeness at the epidemiological level, more samples may be sequenced from each cluster.

The specimens included in the present work were collected in the decreasing phase of the COVID-19 wave that occurred in Tunisia starting from September 2020 and increasing until January 2021. This period was characterized by high transmission within the population, and this explains the high genetic diversity that we found in the sequences obtained. Several lineages were identified, and more than 100 different amino acid changes in comparison to the standard Wuhan strain were identified all through the viral genome.

During the study period, the first isolates of the Alpha VOC, initially identified in the United Kingdom, were detected. The sequenced isolates had the H69del, V70del, Y144del, N501Y, A570D, D614G, P681H, T716I, S982A, and D1118H amino acid substitutions in common with the 20I/501Y.V1 variant (United Kingdom). Thus, it is highly expected that the genetic features described herein will rapidly change to a lower genetic variability and a predominance of the Alpha lineage. Indeed, this is what happened in most countries of the world where the Alpha lineage was introduced, causing devastating waves of COVID-19 (26, 27). With its higher transmissibility within the human population, it becomes rapidly predominant once introduced, and this is also what happened in Tunisia. This work was performed during the decreasing phase of the second wave of COVID-19 and the beginning of the third wave due to the Alpha variant in Tunisia. The country experienced further waves of COVID-19 with higher transmission rates, especially after the emergence of the Delta SARS-CoV-2 variant starting from June 2021, with detrimental impacts on public health and health care systems, and again, we were able to detect, with the same approach, the first isolates of the Delta variant (data not shown) based on the specific mutations of this variant that occur within the sequenced segment: T478K, D614G, and P681R. This target region has allowed the detection of mutations specific to almost all the VOCs and VOIs that have been identified to date, but if a new variant emerges with specific mutations outside the target region, it will be easy to extend or to change the sequenced fragment by simply changing one or two primers.

Furthermore, we were able to detect viruses belonging to the A.27 lineage, initially detected in Denmark and now classified as a VOI. This lineage was detected in about 26 different countries around the world, from Europe to Africa, as well as the United States and Australia. Whole-genome sequencing of three isolates in this series revealed the presence of amino acid substitutions characteristic of this lineage, including L18F, L452R, N501Y, A653V, H655Y, D796Y, and G1219V, and the absence of the D614G substitution in the spike protein. One strain (SP-0347) presented two additional substitutions: P26S, which is found in the Gamma variant (Brazil), and Q677H, found in the Henri Mondor variant detected in different regions of France (28).

We have also detected one sequence (SP-0062, in subcluster 1a) with a mutational profile corresponding to that of the Eta variant, initially detected in Nigeria and in the United Kingdom. This variant had been detected in 48 different countries around the world at the time of writing and is presently classified as a VOI.

The rest of the sequences from subclusters 1b and 1c belonged to the B.1.160 and B.1.177 lineages that are not presently identified as VOCs or VOIs. These sequences exhibit quite high genetic variability, which is expected after the high active-transmission period that the country experienced in late 2020 and January 2021. Among all these variants, some may disappear and others may persist or even dominate if they have a selective advantage in terms of virulence or transmissibility.

### Conclusion.

In conclusion, this study gives an overview of the SARS-CoV-2 strains circulating in Tunisia after a high-transmission wave of COVID-19. Partial S gene sequencing followed by whole-genome sequencing of a selection of specimens was used to identify the different circulating variants. This strategy may be of interest for several countries; it helps to establish a genomic surveillance that is now greatly needed in all regions of the world, with a good cost/effectiveness ratio.

## MATERIALS AND METHODS

### Nasopharyngeal samples.

**FIG 3:**
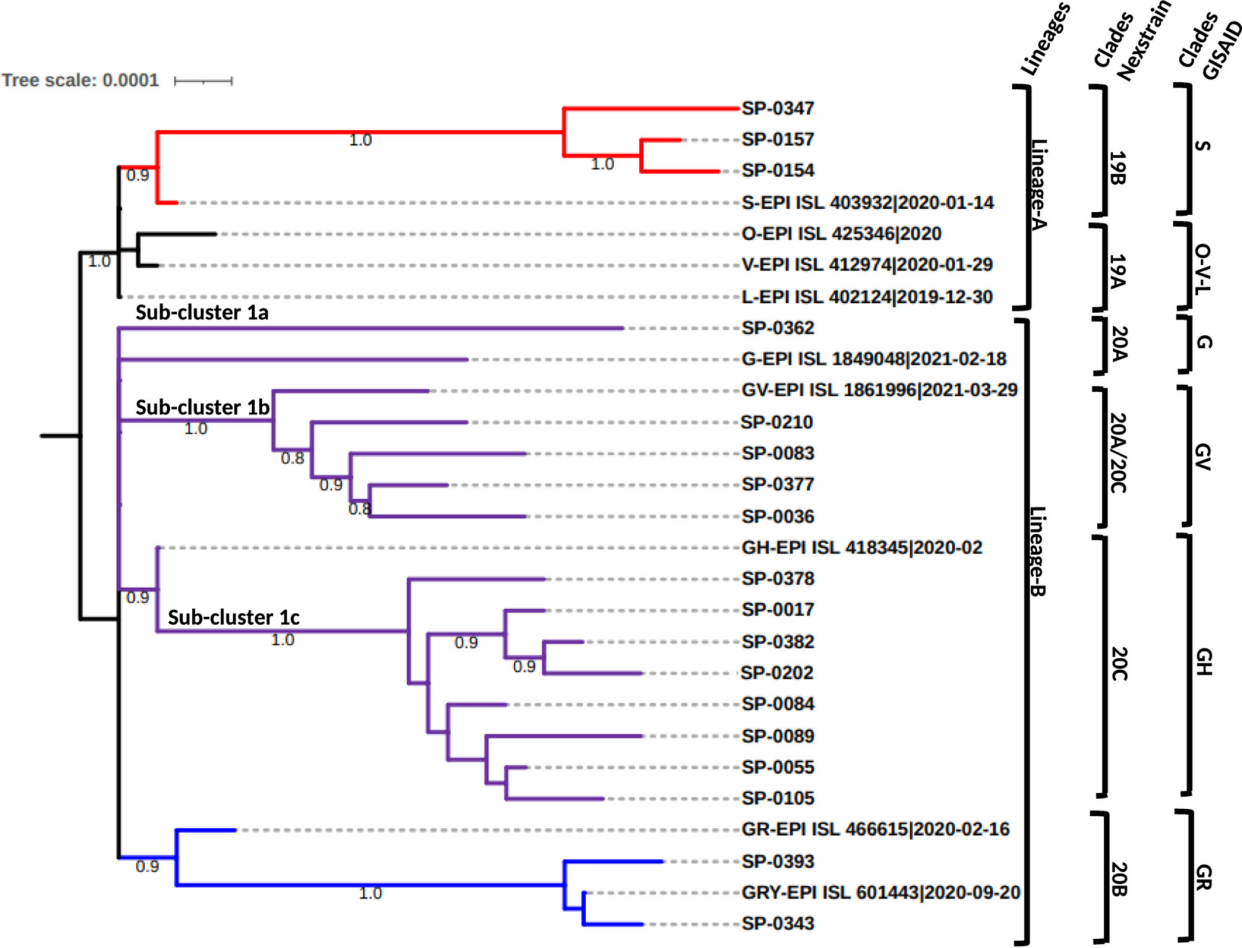
Phylogenetic tree of 18 SARS-CoV-2 whole-genome sequences circulating in Tunisia compared to 9 reference strain genomes. The phylogenetic tree includes 18 Tunisian sequences compared to 9 representative reference sequences of SARS-Cov-2 clades. The tree was constructed using the neighbor-joining method and the Tamura 3-parameter (T92) model. Topology was supported by 1,000 bootstrap replicates. The sequences reported in this study are indicated by the laboratory code. The sequences downloaded from GISAID database are indicated by their accession numbers. Cluster 1 is marked in purple, clade 2 is marked in blue, and clade 3 is marked in red.

A total of 201 SARS-CoV-2-positive nasopharyngeal samples, collected from individuals living in the four districts of Tunis, the capital of Tunisia, were included in this study. Sample collection was performed from January to March 2021, during the decreasing phase of the second wave of the COVID-19 outbreak in Tunisia (Fig. 3). The study population included symptomatic patients presenting with mild COVID clinical forms or with severe forms, as well as asymptomatic individuals sampled after contact with confirmed cases. The study population included 91 males and 110 females, whose ages ranged from 5 to 98 years. The samples were collected by health teams from the Ministry of Health, at home for asymptomatic individuals and those with nonsevere clinical symptoms or at the health facility level for hospitalized patients. Samples were transported, under refrigeration and within 24 h, to the Pasteur Institute of Tunis where they were immediately processed for SARS-CoV-2 detection by specific real-time reverse transcription PCR (RT-PCR) according to WHO-approved protocols (29, 30).

### Ethical statement.

This work was performed in the context of COVID-19 diagnostic efforts, and all samples used for analysis were anonymized. This study was approved by the Bio-Medical Ethics Committee of the Pasteur Institute of Tunis under Tunisia reference no. 2020/14/I/LR16IPT/V1.

### Primer design.

Primers were designed using PrimerDesign-M online software, available through https://www.hiv.lanl.gov/content/sequence/PRIMER_DESIGN/primer_design.html (31, 32), based on an alignment of 13,451 SARS-CoV-2 complete-genome sequences. Several points were considered, such as melting temperatures, G+C percentage, entropy, complexity, and nucleotide composition, in order to perfectly align with the SARS-CoV-2 sequence. The selected primer sequences were as follows: IPT_FW (positions 22964 to 22987), 5′ATTTCAACTGAAATCTATCAGGCC3′, and IPT_REV (positions 23666 to 23647), 5′CTGCACCAAGTGACATAGTG3′. The indicated positions correspond to the sequence of the Wuhan reference strain (accession number NC_045512). The designed primers allow the amplification of a 703-nucleotide-long region in the S gene holding key mutations, including E484K, N501Y, A570D, D614G, and P681H, recently identified as specific to the main VOCs and VUIs of SARS-CoV-2.

### PCR amplification and sequencing in the S gene.

Volumes of 140 μl of nasopharyngeal samples were used for viral RNA extraction with the viral RNA minikit (Qiagen, Hilden, Germany) to give a final elution volume of 60 μl of total RNA. The presence of SARS-CoV-2 RNA was determined by conventional reverse transcription-PCR using the SuperScript III one-step RT-PCR system with the Platinum *Taq* DNA polymerase kit (Invitrogen) in a 25-μl reaction mixture volume containing 12.5 μl of 2× buffer, 0.5 μl of RNasin (Promega), 1 μl each of reverse and forward primers (10 μM), 1 μl of enzyme mix and 5 μl of total extracted RNA. Optimized cycling conditions were performed as follows: reverse transcription with the initial incubation at 50°C for 30 min and 94°C for 2 min, followed by 35 cycles repeating denaturation at 94°C for 15 s, annealing at 54°C for 45 s, and elongation at 72°C for 30 s, and a final elongation at 72°C for 10 min. Amplification products were first visualized by electrophoresis in agarose gels and then purified by the ExoSAP-IT method using exonuclease I and shrimp alkaline phosphatase (Invitrogen). The purified amplicons were sequenced using the BigDye Terminator v3.1 kit (Applied Biosystems) and the forward and reverse PCR primers. The resulting consensus sequences were deduced by aligning the forward and the reverse sequence of each isolate, excluding primer binding regions, and are 659 nucleotides long (positions 22988 to 23665 according to the Wuhan reference strain NC_045512). They were submitted to the NCBI database under accession numbers MZ150010 to MZ150210.

### Whole-genome sequencing.

The QIAseq SARS-CoV-2 primer panel paired with the QIAseq FX DNA library construction kit (Qiagen GmbH, Germany) was used for enriching and sequencing the entire SARS-CoV-2 viral genome. Extracted RNA from nasopharyngeal swabs was first depleted of rRNA using the RiboZero rRNA removal kit (Illumina, USA). The residual RNA was then converted to double-stranded cDNA using random priming. Following cDNA synthesis, the QIAseq SARS-CoV-2 primer panel kit was used, including high-fidelity multiplex PCR yielding 400-bp amplicons covering the full viral genome. The multiplexed amplicon pools were then converted to sequencing libraries by enzymatic fragmentation with a 250-bp fragment size, end repair, and ligation to adapters with the QIAseq FX DNA library construction kit. Thereafter, the constructed DNA library was purified, and adapter dimers were removed with Agencourt AMPure XP beads. The libraries were sequenced using NextSeq (Illumina, Inc., USA) to generate 2 × 150-bp paired-end sequencing reads.

The sequences’ raw data were processed using FastQC version 0.11.9 for quality control (https://www.bioinformatics.babraham.ac.uk/projects/fastqc/). Low-quality reads and adapters were filtered using Trimmomatic version 0.39 (33) with a Phred quality score of 30 as the threshold. Genome consensus sequences were assembled by mapping to the SARS-CoV-2 reference genome with GenBank accession number NC_045512 (Wuhan-Hu-1 isolate) using Spades assembler version 3.15.0 (34), with thresholds of 80% for nucleotide sequence coverage and 90% for nucleotide similarity. The new SARS-CoV-2 sequences obtained were submitted to the GISAID database (https://www.gisaid.org) (35, 36) with the following accession numbers: EPI_ISL_2035560, EPI_ISL_2035563, EPI_ISL_2035720, EPI_ISL_2035734, EPI_ISL_2035752, EPI_ISL_2035753, EPI_ISL_2035940 to EPI_ISL_2035949, EPI_ISL_2035988, and EPI_ISL_2036077.

### Phylogenetic analysis.

The obtained partial S gene sequences and selected whole-genome sequences were aligned with representative SARS-CoV-2 reference sequences of the nine recognized GISAID clades publicly available in the GISAID database by using MUSCLE multiple sequence alignment algorithms (37) implemented in MEGAX (38). Phylogenetic analyses were performed on nucleotide sequences using the maximum-likelihood method with the Tamura 3-parameter model and then on amino acid sequences, obtained from the aligned sequences, using the maximum-likelihood method and the Jones-Taylor-Thornton model. The tree topologies were supported by 1,000 bootstrap replicates.

Mutation profiles in the open reading frame 1a (ORF1a), ORF1b, S, ORF3a, E, M, ORF6, ORF7a, ORF8, N, and ORF10 genomic regions of SARS-CoV-2 were assessed by comparing the nucleotide and deduced amino acid sequences of the Tunisian strains with those of the Wuhan reference strain, using the sequence alignment performed by MUSCLE multiple sequence alignment algorithms (37) implemented in MEGAX (38).

### Data availability.

Partial S gene amplicon consensus sequences were submitted to the NCBI database under accession numbers MZ150010 to MZ150210. New SARS-CoV-2 sequences generated here were submitted to the GISAID database (https://www.gisaid.org) (35, 36) with the following accession numbers: EPI_ISL_2035560, EPI_ISL_2035563, EPI_ISL_2035720, EPI_ISL_2035734, EPI_ISL_2035752, EPI_ISL_2035753, EPI_ISL_2035940 to EPI_ISL_2035949, EPI_ISL_2035988 and EPI_ISL_2036077.
